# Exploring the associations of cortical connectivity-modulation with clinical and psychological measures in acute low back pain

**DOI:** 10.3389/fpain.2026.1884788

**Published:** 2026-08-27

**Authors:** Sabina Hotz-Boendermaker, Bart Boendermaker, Roman Buechler, Lars Michels

**Affiliations:** 1 School of Health Sciences, Institute of Physiotherapy, University of Applied Sciences ZHAW, Winterthur, Switzerland; 2 Department of Human Physiology and Physiotherapy, Faculty of Physical Education & Physiotherapy, Vrije Universiteit Brussel, Brussels, Belgium; 3 Department of Physical Medicine and Physiotherapy, University Hospital Brussels, Brussels, Belgium; 4 Department of Neuroradiology, University Hospital Zurich, Zurich, Switzerland; 5 Neuroscience Center Zurich, University of Zurich and Swiss Federal Institute of Technology Zurich, Zurich, Switzerland

**Keywords:** connectivity, fear avoidance, functional MRI, low back pain, movement control, wind-up ratio

## Abstract

**Background:**

Low back pain (LBP) represents a significant global health burden. Transitioning from the traditional “self-limiting” perspective, emerging evidence suggests that neural plasticity and central maladaptation in the acute phase are pivotal in the progression to chronicity.

**Objectives:**

This study investigated whole-brain functional connectivity and sensorimotor processing during acute LBP.

**Methods:**

Thirteen participants with acute LBP (<4 weeks duration) underwent clinical, psychometric, and functional magnetic resonance imaging (fMRI) assessments. Brain activity and connectivity were mapped during lumbar mechanosensory stimulation, with a focused analysis on sensorimotor, salience, cerebellar, and limbic networks.

**Results:**

Pain intensity demonstrated a negative correlation with posterior supramarginal-precuneus connectivity and a positive correlation with cerebellar activity. Fear-avoidance behavior was positively associated with connectivity across a cerebellar-limbic axis (cerebellum, brainstem, and hippocampus). Furthermore, mechanical pain summation (wind-up ratio) and movement control impairments were significantly linked to connectivity shifts within the salience and sensorimotor-lateral networks.

**Conclusion:**

These findings demonstrate that acute LBP is characterized by immediate widespread neuronal reorganization. The modulation of these networks by pain intensity and motor dysfunction suggests an early interplay between sensory, motor, and affective processing. Identifying these neural signatures provides a mechanistic framework for targeted biopsychosocial interventions designed to intercept the transition from acute pain to chronic disability.

## Introduction

Low back pain (LBP) is a widespread global health problem with a significant medical, economic, and social burden (1). While acute LBP has historically been considered a self-limiting condition, recent evidence challenges this assumption (2). Although many individuals experience symptom relief within weeks, a significant proportion continues to report mild to moderate LBP for months, with a high rate of relapse within the first year (3, 4). This raises critical questions about the factors contributing to the transition from acute to persistent pain. While structural and tissue damage play a role in the early stages, they do not fully explain why pain persists in many cases after tissue healing (5, 6). Increasingly, researchers are turning their attention to the nervous system, particularly the roles of neural plasticity and central processing mechanisms in response to painful stimuli, which may contribute to the maintenance of pain persistence (7, 8). Neuroscience research, including functional neuroimaging studies, has shown changes in brain activity and connectivity, particularly in regions associated with pain perception, emotional regulation, and sensorimotor control (9–11). These findings suggest that LBP is not simply the result of peripheral damage but involves complex changes across multiple brain networks (12). These processes further complicate pain management in individuals with LBP (5). Unfortunately, previous studies have mainly focused on persistent pain, whereas investigation in the acute state may help prevent secondary chronicity of LBP (13).

This neuroimaging study formed part of a 12-month prospective cohort study investigating psychometric and clinical variables in acute LBP. We have shown that nearly half of the participants experienced less favorable pain trajectories (3). We also demonstrated evidence of adverse long-term effects of baseline pain, activity persistence behavior, and wind-up phenomena (14, 15). However, the relationship between these biopsychosocial variables and brain network functioning needs to be investigated. Previously, using functional magnetic resonance imaging (fMRI) in subjects with acute LBP, we identified early adaptive changes in sensorimotor processing, as evidenced by connectivity within salience, cerebellar, and limbic networks (16). These neuronal changes may provide insight into the multiple consequences of acute LBP, particularly regarding anticipatory postural control.

This study aimed to explore the associations between adaptive sensorimotor processing in the brain and psychometric and clinical assessments in individuals with acute LBP. The findings may shed light on the transition from acute to persistent symptoms by identifying how neural adaptations in the early stages of acute pain are related to clinical outcomes. A refined understanding of the relationship between brain network connectivity and biopsychosocial variables could lead to improved treatment strategies and a reduction in the burden of LBP on individuals and society.

## Materials and methods

### Subjects

This neuroimaging study formed part of a large-scale, prospective cohort study investigating the clinical and psychosocial factors associated with acute LBP (17). Thirteen participants with acute LBP were included in the data analysis (8 female, mean age 33.46 ± 13.34 years, range 18–60). Participants with LBP were recruited from physiotherapy practices, hospital outpatient departments, and a university campus via word of mouth, advertisements, and mailing lists. Inclusion criteria were LBP for less than four weeks and being pain-free for the previous six months. Exclusion criteria were any clinically relevant anatomical abnormality of the lumbar spine (e.g., fracture, carcinoma), had undergone spinal surgery, had peripheral or central neurological disease, major psychiatric disorder, were pregnant, or had any other condition that would prevent them from participating in MR imaging. The study was approved by the Ethics Committee of the Canton of Zurich, Switzerland (BASEC no. 2016-02096) and was conducted in accordance with the Declaration of Helsinki. Participants were compensated for their involvement with traffic spence and a voucher.

### Experimental procedure

Within one week, participants with LBP had to complete an online survey assessing demographic, clinical, and psychometric data, a clinical assessment, and an MRI scan. Data were collected via an online survey (SurveyMonkey). A link was emailed, and participants were asked to complete the questionnaires (3).

#### Online survey with psychometric questionnaires

- A numerical rating scale (NRS) was used to measure pain intensity (18). The scale ranges from 0 to 10, with 0 representing «no pain» and 10 representing «pain as severe as you can imagine.» Pain intensity was calculated as the mean of actual, average, and maximum perceived pain over the previous 2 weeks (3).
- The Oswestry Disability Questionnaire (ODI) assesses functional disability due to LBP (19). The ten items are scored on a 6-point Likert scale (0–5, with 5 indicating the highest disability). The index was calculated by dividing the total score by the maximum score (50 points) and multiplying by 100. A higher index indicates greater disability (20).
- The 21-item Depression Anxiety Stress Scale (DASS) questionnaire was designed to assess the severity of depression, anxiety, and stress using three subscales (21). Only the depression subscale was used in the present study. The subscale score was calculated independently and then doubled to reflect the short version of the questionnaire. Depressive symptoms have a cut-off score of >10 on the depression subscale (21).
- The Avoidance-Endurance Questionnaire measures pain-related cognitive, affective, and behavioral responses (22). The persistence subscale contains seven items, while the avoidance subscale contains 11 items. Each item was measured on a 7-point Likert scale (0–6, with 6 indicating always). Each subscale's mean was computed, with higher scores indicating greater avoidance or persistence behavior.

#### Clinical assessment

Clinical examinations were performed within one week by experienced physiotherapists who received two hours of training.

- Temporal summation of somatosensory stimulation was tested using a toothpick to apply pinpricks bilaterally at the L4 level (14). After two and ten stimulations with a one-second interval between stimuli, participants rated pain intensity on an NRS. In the end, the wind-up ratio was calculated as the ratio in the pain intensity of the stimulus series. For the calculation of the WUR, mathematically invalid values can occur if the subject indicates 0 on the NRS, i.e., a painless stimulation during the first stimulation. The following mathematical approximation to the WUR formula solved this dilemma: painintensityafter10 stimulations + 1/ painintensityafter1stimulation + 1 (14).
- Movement control of the lower back was quantified using the Movement Control Impairment (MCI) Test (23). The test battery includes six active tests involving extension, flexion, and lateral flexion [waiter's bow, pelvic tilt, one-legged stance, sitting knee extension, quadruped pelvic transfer (rocking forward and rocking backward), and prone active knee flexion]. The test score is based on the number of positive (i.e., incorrectly performed) tests. The study's cut-off score for confirming MCI was two positive tests (24).

### Neuroimaging data acquisition and analysis

#### Lumbar spine paradigm

All participants were scanned in the prone position. Lumbar stimulation consisted of non-painful posterior to the anterior intervertebral movement (PA) of the lumbar spinous processes L1 and L4 by the examiner's thumb (16, 25). The stimulation induced a small physiological motion in a functional spinal unit (FSU), with biomechanical characteristics similar to those of the whole spine. MR-compatible pressure force sensors were attached to the back and controlled for the pressure force of 30 Newtons, which was then converted into an appropriate voltage signal (25). The event-related fMRI experiment consisted of 17 stimuli, each 5 s in duration, with a randomized inter-stimulus interval of 8–10 s for the L1 and L4 stimulation levels. The start and end of the stimulus and the load appeared on a screen in front of the experimenter in the MRI room.

#### Neuroimaging data acquisition and analysis

MRI data were acquired at the University Hospital of Zurich using a Philips Ingenia 3T whole-body MR unit with a 32-channel head coil. Task-based functional scans consisting of 270 functional images (540 s) included a sensitivity-encoded single-shot (factor 41) T2*-weighted echoplanar imaging (EPI) sequence. Parameters were set as follows: repetition time [TR] = 2,000 ms, echo time [TE] = 30 ms, field of view [FOV] = 220 mm×220 mm, acquisition matrix size in plane = 72 × 74, interpolated to 128 × 128, 36 slices (slice thickness = 3 mm, inter-slice gap = 0.6 mm) with a spatial resolution of 3 × 3 × 3 mm^3^ [reconstructed 1.72 × 1.72 × 3 mm^3^], flip angle = 78°, and sensitivity-coded acceleration factor R = 1.8.

We placed the contiguous axial slices along the anterior-posterior commissural plane on a midsagittal scout image, covering the entire brain, including the cerebellum. Slices were acquired in ascending order. Three dummy scans were acquired first to achieve steady-state magnetization and were subsequently discarded. We also acquired 3-dimensional T1-weighted anatomical images [160 slices; TR = 8.1 ms; TE = 3.7 ms; flip angle = 8°; FOV = 240 × 240 mm^2^; spatial resolution 1 × 1 × 1 mm^3^ (reconstructed 0.94 × 0.94 × 0.94 mm^3^)].

Imaging data analysis was performed using the SPM12 (Wellcome Department of Imaging Neuroscience, London, UK, http://www.fil.ion.ucl.ac.uk/spm) and CONN toolbox (version 18. a) software packages (26) running on MATLAB R2019a (Mathworks Inc, Sherbon, MA). Pre-processing of the fMRI data consisted of the following steps: realignment, slice-timing correction, coregistration to the structural T1-weighted image, segmentation, spatial normalization to the Montreal Neurological Institute (MNI) coordinate space, and spatial smoothing (Gaussian kernel with a full width at half maximum of 6 mm). The pre-processed data were imported into the CONN toolbox (version 18.a) (27) to perform the following processing steps: filtering (0.01–0.1 Hz), denoising, first- and second-level analysis. Data from three participants showed excessive head motion during data acquisition (linear shift >3 mm over the whole run and >1.5 mm frame-by-frame, rotation >1°).

The first-level analysis included two conditions, i.e., stimulation and no stimulation (rest) as the baseline. The stimulation condition was characterized by pressure applied to the lumbar vertebrae (L1 and L4, respectively) for 5 s, and the absence of pressure defined the baseline. Using a whole-brain seed-to-voxel approach, we performed generalized psychophysiological interaction (gPPI) analyses, i.e., the modulation of functional connectivity by stimulation (relative to rest). The two main task conditions (i.e., stimulation and rest), the six motion parameters, and the white matter and cerebrospinal fluid noise components were included in the model to regress out potentially confounding effects. In addition, temporal band-pass filtering (0.008 Hz < f < 0.09 Hz) was applied to remove high-frequency fluctuations and minimize the effects of noise (e.g., physiological activity), and linear detrending was applied.

The seeds of the functional networks studied were selected based on our prior outcomes (16). They were defined by the FSL Harvard-Oxford atlas as implemented in the CONN toolbox (26): The *salience network* with frontal orbital cortex, rostral prefrontal cortex; insular cortex; supramarginal gyrus, anterior and posterior division, and the anterior division of the cingulate cortex as seeds; the lateral and the superior *sensorimotor networks*; *the limbic network* comprising the parahippocampal gyrus, anterior and posterior division; hippocampus; brainstem and amygdala as seeds; and the *anterior and posterior cerebellar* networks. Based on regression analysis (bivariate), functional seed-to-voxel maps of connectivity modulation were generated for each subject. The resulting beta-value reflects the difference between beta values (= slope) during stimulation and rest (beta_stimulation–beta_rest = Δβ). The significant clusters show brain regions whose connectivity to the seed ROI differs significantly between stimulation and rest. Specifically, the Δβ reflects the BOLD signal change in the target ROI per unit change in BOLD signal in the seed ROI during stimulation relative to rest.

#### Association of connectivity-modulation with psychometric data and clinical assessments

**Figure 1:**
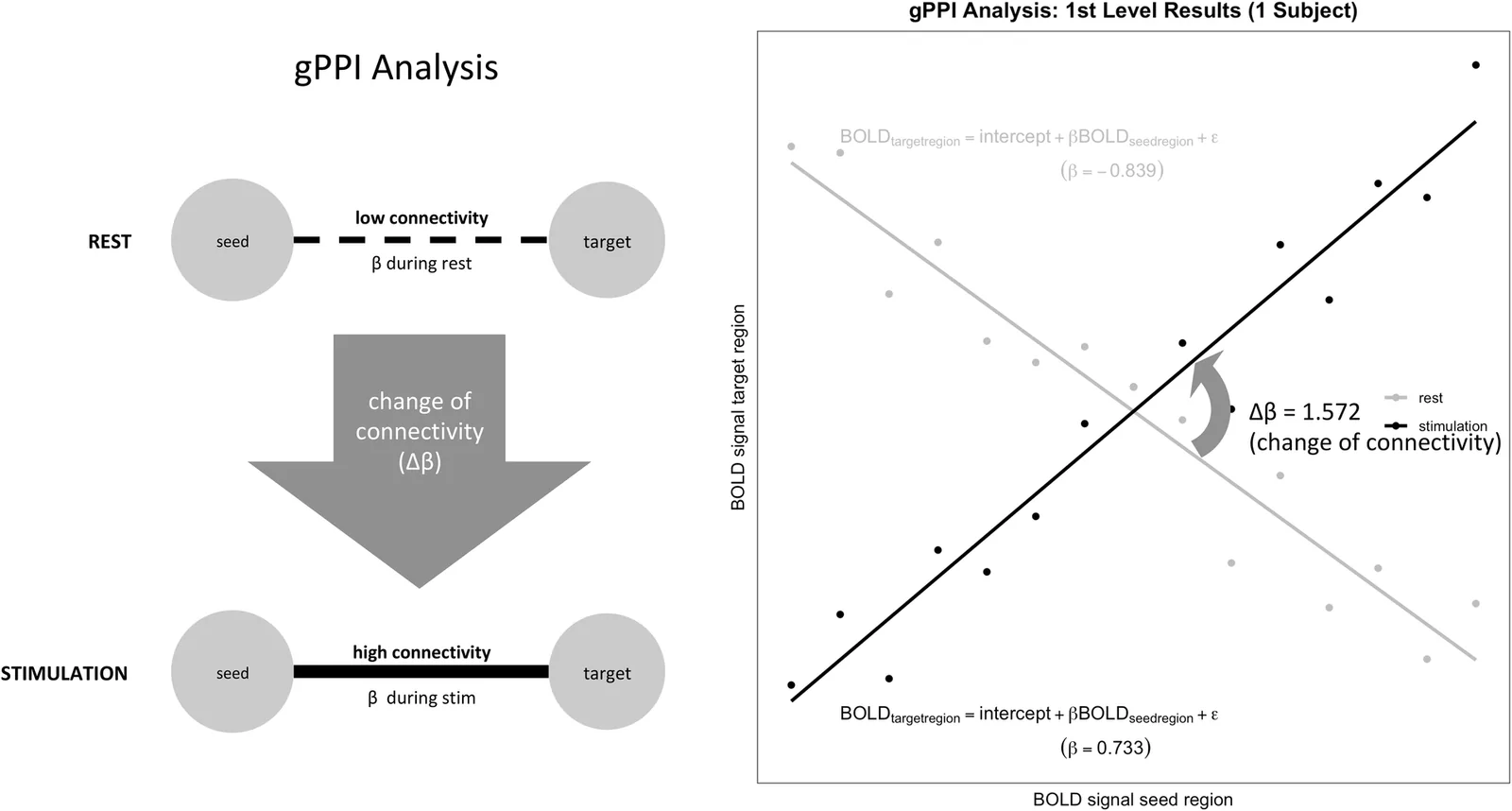
Display of the predictor and connectivity-modulation. The *Δβ* value derived from the first-level analysis as the dependent variable.

The second-level analyses assessed the associations of connectivity-modulation (Δβ) during stimulation) and the psychometric/clinical data. The beta values reported reflect the slope of the regression analysis (clinical/psychometric variable as the predictor and connectivity-modulation (Δβ value from the first-level analysis as the dependent variable, see Figure 1).

From the online survey, we used the following psychometric measures: pain intensity, avoidance, and persistence behavior scales. As the depression scale and disability index did not produce any clinically relevant values, they were not included in the analysis. The clinical assessments included the MCI score and the wind-up ratio (right side). In the CONN Toolbox, the effect size is represented by these beta values, which quantify the connectivity between the relative change (stimulation–rest) and the clinical variables. These values measure the magnitude of the connectivity change per unit change in the respective variable. We used the nomenclature from the Harvard-Oxford Atlas to label the anatomical localization of significant activation peaks (28, 29).

## Results

The online survey collected socio-demographic and psychometric data. These baseline characteristics of the participants are shown in Table 1. The acute LBP participants reported a mean pain intensity of 4.5 on the NRS. Individual participants ranged from mild to severe. In contrast, the LBP-associated disability index showed a mild mean disability of <20%, and the range did not extend to a moderate level of <40%.

**Table 1:** Baseline characteristics of the psychometric data and clinical assessments.

Online Survey questionnaire *N* = 13	Mean (Standard Deviation)	Range
Pain intensity (NRS)	4.7 (1.2)	3.5–7.5
LBP associated disability (ODI Index) in %	12.1 (7.5)	0–31
Depression Anxiety Stress Scale		
*Depression subscale*	2.4 (2.6)	0–7
Avoidance Endurance Questionnaire		
*Avoidance subscale*	3.4 (1.5)	0.4–6
*Persistence subscale*	3.2 (0.9)	2–5.1
Clinical Assessment *N* = 13		
Movement control impairment test score	2 (1.3)	0–5
Wind-up ratio (temporal summation),		
*Wind-up ratio left side*	1.8 (0.8)	1–3.5
*Wind-up ratio right side*	1.5 (0.7)	0.8–3

NRS, Numeric rating scale, ODI index, Oswestry disability index.

The clinical assessment consisted of tests of trunk movement control and temporal summation, calculated as the wind-up ratio (Table 1). The movement control test battery showed a mean of two positive (i.e., inaccurately performed) tests, with a range of 0–6. There was evidence of impaired movement control, with at least two positive tests among 9 participants. Temporal summation, computed as the wind-up ratio, was slightly greater on the left side, and the scores showed substantial inter-participant variation.

### Associations of functional connectivity-modulation with psychometric and clinical data

Whole-brain analysis was used to explore the relationship between the strength of functional connectivity-modulation and psychometric data and their respective clinical assessments, as shown in Table 2.

**Table 2:** Correlation of PPI beta weights (Delta rest—stim) with questionnaire scores and clinical parameters.

**Network**	**Seed**	**Brain regions**	**Beta Weight**	**Left x,y,z**			**Right x,y,z**			**Cluster size**
Pain Intensity										
*Salience network*	Supramarginal gyrus, posterior division _R	Precuneus cortex	0.27	−24	−82	34				70
		Cuneal cortex	0.24				10	−66	8	260
		Precuneus cortex	0.21	−30	−72	46				59
*Sensorimotor network*	Parietal operculum cortex_L	Cerebellum VI	0.51				18	−74	−22	57
*Limbic network*	Parahippocampal gyrus, posterior division _L	Inferior frontal gyrus	−0.61	−52	24	−4				106
		Cingulate gyrus, anterior division	0.54	−2	44	−2				50
	Parahippocampal gyrus, posterior division _R	Middle temporal gyrus, posterior division	−0.52	−66	−40	−8				107
	Parahippocampal gyrus, anterior division _L	Cerebellum VI	−0.20				16	−54	−30	119
Avoidance behavior										
*Salience network*	Cingulate gyrus, anterior division _R	Postcentral gyrus	−0.27				12	−32	64	101
*Limbic network*	Hippocampus_R	Amygdala/parahippocampal gyrus	0.41	−34	−6	−30				129
		Inferior temporal gyrus, posterior division	0.38	−55	−22	−26				77
		Middle frontal gyrus	0.31	−30	20	34				95
		Cerebellum VI	0.28				4	−56	−14	69
		Cerebellum IV	−0.27	−14	−44	−28				116
		Inferior temporal gyrus, anterior division	0.27	−46	−6	−34				105
	Amygdala_R	Temporal fusiform cortex, anterior division	0.30	26	−6	−46				74
		Inferior temporal gyrus, posterior division	0.25	−38	−2	−48				646
		Inferior temporal gyrus, anterior division	0.25	−60	−48	−14				245
*Salience network*	Supramarginal gyrus, anterior division_R	Frontal Operculum/ Insular cortex	0.33	−52	−14	20				
	Middle temporal gyrus, anterior division									
	Supramarginal gyrus, posterior division_R	Brainstem	−1.09	−2	−28	−18				76
	Insular cortex_R	Superior/middle frontal gyrus	−0.47				24	20	58	198
	Insular cortex_L	Middle temporal gyrus	0.45	−62	−66	0				90
*Limbic network*	Hippocampus_R	Superior frontal gyrus	0.51	−20	16	62				108
	Hippocampus_L	Supramarginal gyrus, posterior division	0.27				42	−42	12	100
	Parahippocampal gyrus, anterior division_L	Lateral occipital cortex, inferior division	0.23				48	−70	2	90
Movement control impairment test										
*Salience network*	Insular cortex_L	Angular gyrus	0.31				46	−58	26	97
	Supramarginal gyrus, anterior division_L	Insular cortex	−0.3				48	4	−6	128
	Supramarginal gyrus, anterior division_R	Supplementary motor area	0.25	−16	10	46				
*Sensorimotor network*	Precentral gyrus L	Supramarginal gyrus, posterior division	0.57	−54	−50	30				60
		Angular gyrus	0.5	−46	−68	22				70
		Lateral occipital cortex	0.42				44	−70	26	68
*Limbic network*	Hippocampus_L	Brainstem, Cerebellum IX	0.53	−4	−38	−40				63
		Frontal pole	−0.46	−22	52	26				130
		Central operculum	−0.31	−46	−8	8				64
		Lateral occipital cortex, superior division	−0.29	−38	−76	14				168
		Lateral occipital cortex, superior division	−0.28				26	−90	26	129
	Parahippocoampal gyrus, posterior division_R	Angular gyrus	−0.24	−46	−44	30				80
	Parahippocoampal gyrus, posterior division_L	Precentral gyrus	−0.25	−58	6	18				75

### Psychometric data

#### Pain intensity

**Figure 2:**
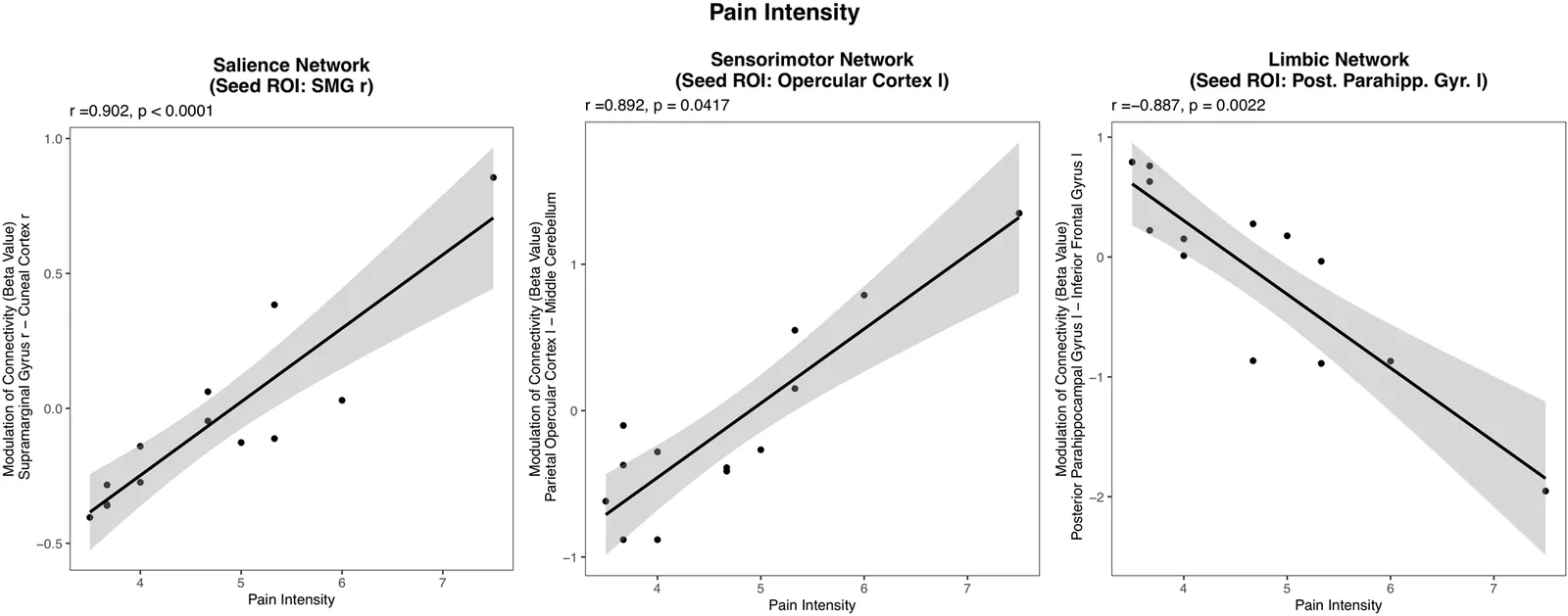
Association between stimulation-induced connectivity modulation and pain intensity.

In the *salience network*, pain intensity was moderately associated with the right supramarginal gyrus, posterior division (seed), and different clusters in the cuneal and precuneus cortex (*β* = 0.27–0.21). In the *sensorimotor lateral network*, pain intensity was strongly linked with the middle cerebellum (seed left parietal operculum cortex_L, *β* = 0.51), see Table 2, Figure 2.

Finally, in the *limbic network,* strong negative associations were computed between the posterior division of the parahippocampal gyrus (seed) and the inferior frontal gyrus (*β* = −0.61) and a positive association with the anterior cingulate gyrus (*β* = 0.54). The same seed on the right side was negatively associated with the middle temporal gyrus, posterior division (*β* = −0.52). The seed left parahippocampal gyrus, anterior division, was negatively associated with the middle cerebellum (*β* = −0.20).

#### Avoidance of physical activities

**Figure 3:**
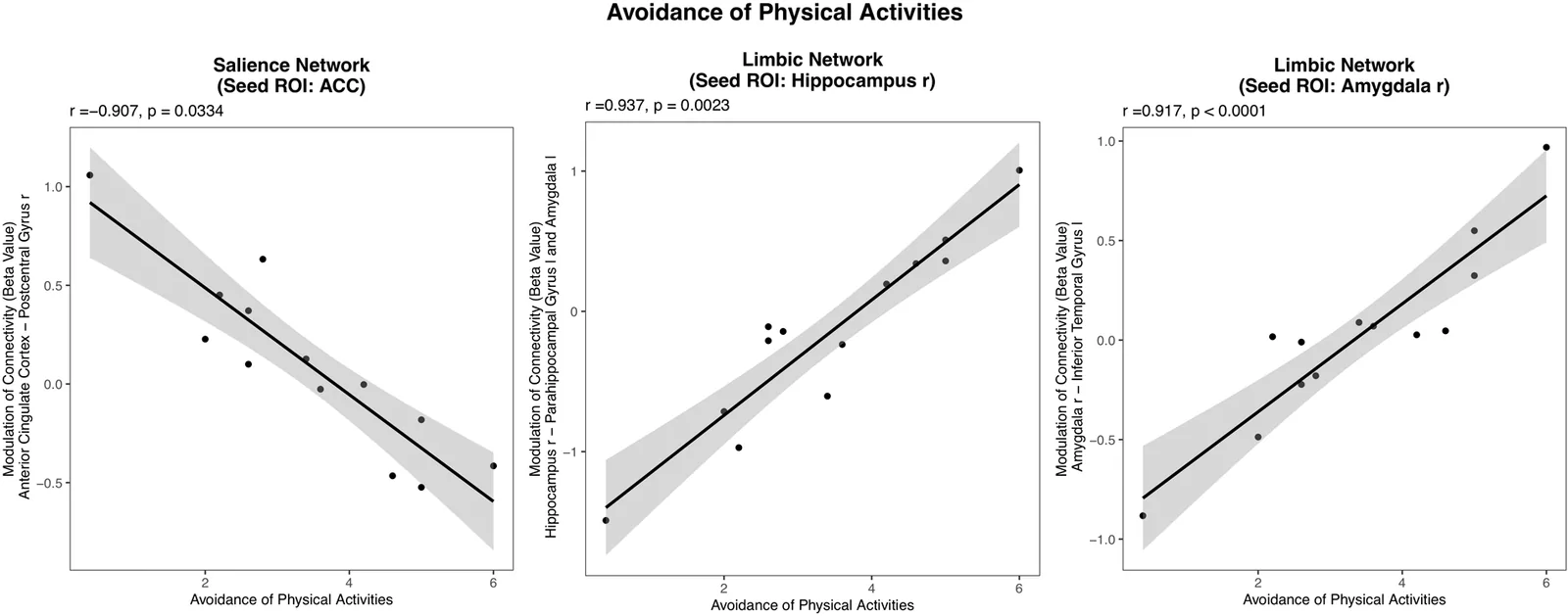
Association between stimulation-induced connectivity modulation and avoidance of physical activities.

Avoidance of physical activity was associated with the *salience* and *limbic networks,* see Table 2 and Figure 3. There was a low negative association between the seed right cingulate gyrus, anterior division, and the postcentral gyrus (*β* = −0.27). The right hippocampus was associated with the amygdala (*β* = 0.41), the anterior and posterior inferior temporal gyri (*β* = 0.38), the middle frontal gyrus (*β* = 0.31), and the right lateral cerebellum (*β* = 0.28) and the left medial cerebellum (*β* = −0.27). The right amygdala was associated with the inferior temporal gyrus, anterior and posterior divisions (*β* = 0.25), and the temporal fusiform cortex (*β* = 0.30).

### Clinical assessments

#### Wind-up ratio on the right side

**Figure 4:**
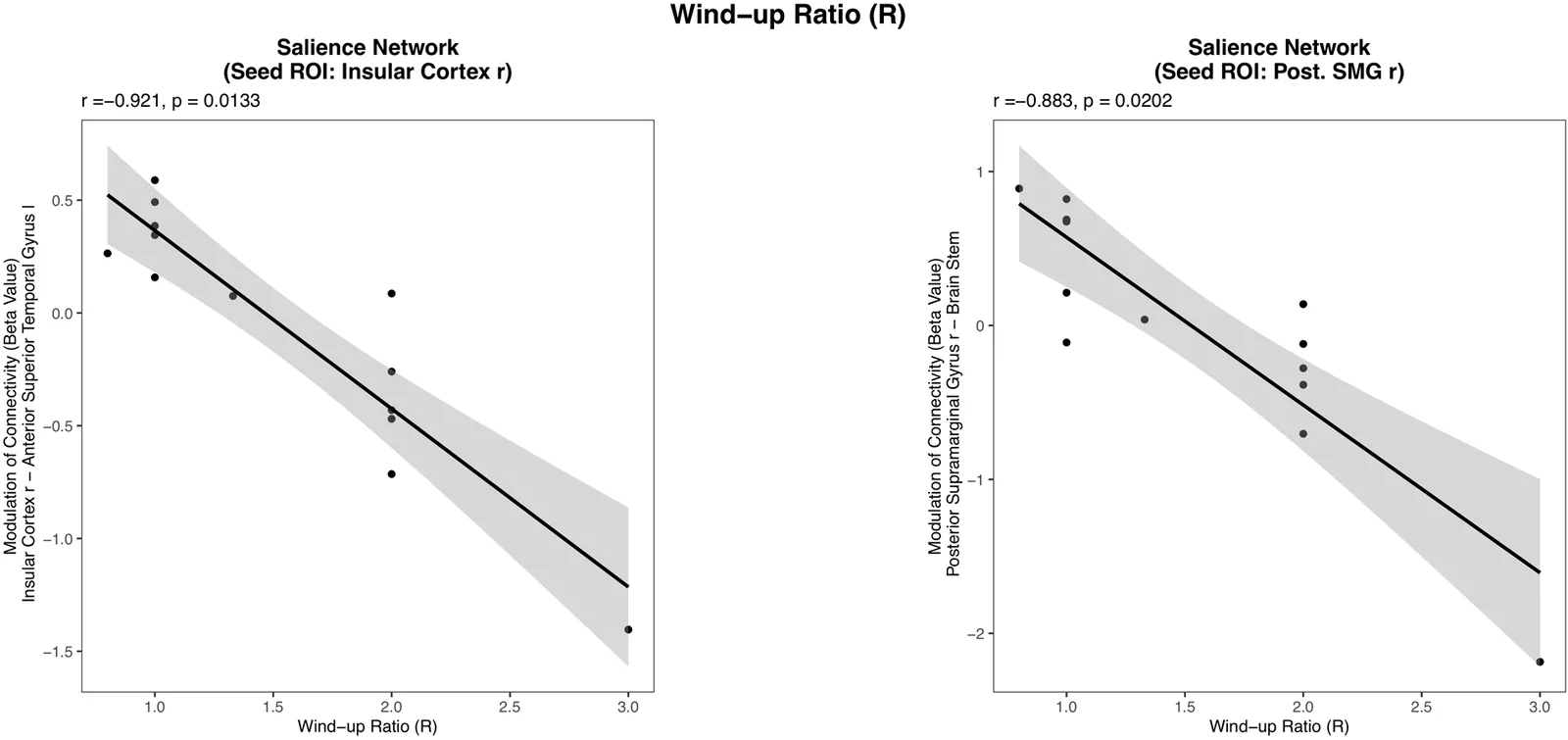
Association between stimulation-induced connectivity modulation and wind−up ratio on the right side.

Within the *salience network*, pinprick temporal summation, computed as the wind-up ratio, was moderately positively associated with operculo-insular cortices. The seed at the right side was negatively associated with the superior/middle frontal gyrus (*β* = −0.47). On the contralateral hemisphere, the same seed was positively correlated with the middle temporal gyrus (*β* = 0.45). The connectivity-modulation between the right supramarginal gyrus, posterior division (seed), and the brain stem was strongly negatively associated with the wind-up ratio (*β* = −1.09). Within *the limbic network,* a positive relation with the left superior frontal gyrus (seed right hippocampus, *β* = 0.51) was found; the seed left hippocampus was associated with the bilateral supramarginal gyrus (*β* = 0.27). The left parahippocampal gyrus was positively connected with the lateral occipital cortex (*β* = 0.23), see Table 2 and Figure 4.

#### Movement control impairment

**Figure 5:**
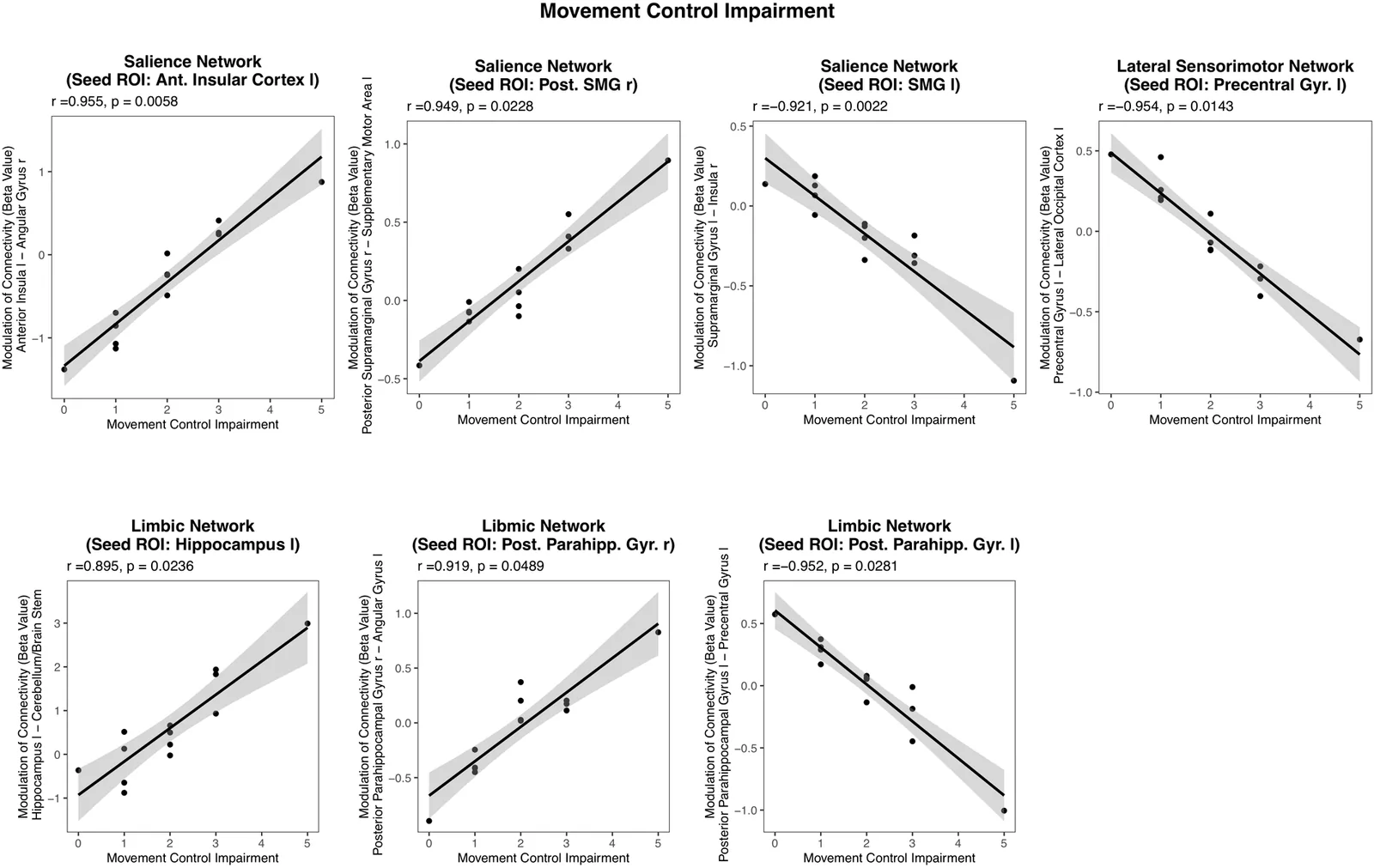
Association between stimulation-induced connectivity modulation and movement control impairment.

The associations for the total score of the movement control impairment test were connected to the operculo-insular, supramarginal, hippocampal, and cerebellar regions, see Table 2 and Figure 5. Within the *salience network*, movement control impairment was positively correlated with connectivity-modulation between the angular gyrus and the left insular cortex (seed, *β* = 0.31) and between the right supramarginal gyrus (seed) and the supplementary motor area (*β* = 0.25). In contrast, the left supramarginal gyrus seed on the opposite side was negatively correlated with the insular cortex (*β* = −0.3). Within the *sensorimotor lateral network,* movement control was moderately associated with the connectivity-modulation between the left precentral gyrus (seed) and the bilateral lateral occipital cortices (*β* = 0.5 and 0.42), as well as with the supramarginal gyrus (*β* = 0.57). Finally, a positive relationship with the left medial cerebellum/brainstem and the left hippocampus (seed; *β* = 0.53) was found in the *limbic network.* Additionally, there were negative associations between the left hippocampus seed and the lateral occipital cortex bilaterally (*β* = −0.29 and −0.28), the central operculum (*β* = −0.31), and the frontal pole (*β* = −0.46). There were negative associations between MCI scores and right parahippocampal gyrus with the angular gyrus (*β* = −0.24), and left parahippocampal gyrus with the precentral gyrus (*β* = −0.25).

## Discussion

This study examined the relationship between functional connectivity-modulation and psychometric and clinical data collected from individuals with acute LBP. The results revealed associations with psychometric data, the wind-up ratio, and MCI tests. These findings point to a relationship between sensorimotor processing and somatic and cognitive-behavioral.

processes in individuals with acute LBP. These processes may prevent further tissue damage and pain sensation; in the long term, they may mediate the persistence of pain.

### Psychometric data

Early connectivity changes have been proposed to describe the adaptive processing of non-painful mechanosensation in acute low back pain (LBP) (16). This study revealed links among brain connectivity-modulation, pain intensity, and avoidance behavior.

#### Pain intensity

Minimal lumbar spine movement induced by mechanosensory stimulation facilitates anticipatory postural control (16). In individuals with acute LBP, the *salience network* acts as a filter to detect behaviorally relevant stimuli while coordinating neural resources in response to cortical input (30, 31). Thus, during trunk movements, nociceptive processing takes precedence over mechanosensory input (32). This prioritization may be motivated by the need to protect the lumbar spine from potential further tissue damage (33). In this context, connectivity changes between the supramarginal and precuneus cortices may indicate anticipation of pain or danger during the induced lumbar spine movements. There is evidence that the involvement of these brain areas may increase the perception of pain as a threat, causing pain-related distress and mediating pain persistence (34). We assume that during the experiment, intrinsic pain was present to some extent, even though no painful stimulation was applied. Therefore, the positive association between these cortical connectivity's and pain intensity suggests that subjects experiencing higher pain levels may be more likely to anticipate pain, which could create a vicious circle.

Within the *sensorimotor network*, the strong relationship between pain intensity and brain networks responsible for somatosensory processing and trunk movement coordination, i.e., the parietal operculum and the middle cerebellum, underscores the interplay between pain intensity and adaptive motor responses (35, 36). Also, the findings within the *limbic network* support these assumptions. In individuals with acute LBP, evidence indicates early associative learning between pain intensity and mechanosensory input during lumbar spine movements (13). The results further deepen our understanding of how assessment of acute LBacute-phase pain intensity may mediate pain persistence. In particular, as our own and others' findings indicate that the intensity of pain experienced during an acute episode is a potent mediator of pain persistence (34). From a clinical perspective, reliable early pain management, as recommended by guidelines for acute LBP, may prevent further maladaptive system responses (2).

#### Avoidance behavior

Neuroimaging studies have demonstrated an association between avoidance behavior and the limbic system, particularly the right amygdala, in individuals with high kinesiophobia, both with and without persistent LBP (37, 38). The study results extend these findings to acute LBP, revealing a moderate association between self-reported avoidance behavior and connectivity-modulation within the *salience* and *limbic networks*. Functional connectivity was observed between the seed regions (hippocampus and amygdala) in the right hemisphere, the frontal and temporal cortices, and the cerebellum. These brain areas are involved in the neural circuits of motivational and avoidance behavioral responses. Thus, these findings reflect contemporary views on the interaction between the mind and body in LBP (39).

### Clinical assessment

#### Wind-up ratio

The wind-up phenomenon involves a progressive increase in pain perception over time in response to repeated mechanosensory stimuli at a constant frequency. It results from amplified peripheral input in the dorsal horn, which may contribute to secondary hyperalgesia and central sensitization in LBP (40). This intensified pain signaling increases activity in the brain's salience network, thereby enhancing the perception and experience of pain and mechanosensation (31, 41). Using a seed ROI in the salience network, connectivity-modulation associated with the wind-up ratio has been found primarily in the operculo-insular region, the supramarginal gyrus, and the frontal cortex. Reduced connectivity modulation within the *salience network* has been demonstrated in these brain areas in individuals with acute LBP compared to a control group (16). The operculo-insular cortex is an integrative hub within larger brain networks, primarily linking advanced somatosensory processing with body awareness and emotional processing via its extensive connections with subcortical and cortical structures (42). Its strong negative association with frontal regions indicates reduced sensory evaluation processes, such as exaggerated threat perception and impaired multisensory prediction and integration, which amplify input (43). The supramarginal gyrus also processes higher-order mechanosensory information. However, it coordinates and prepares trunk movements by acting as a central point that evaluates differences between afferent sensory information and the expected input (44). Its strong negative relationship with the brainstem may indicate the involvement of the autonomic nervous system or stress responses (45). These responses include increased muscle tone, vasoconstriction, and peripheral and central sensitization. All of these factors are crucial yet often overlooked factors in the assessment of acute LBP (46, 47). The frontal gyrus is involved in the top-down cognitive control of sensory experiences, particularly pain perception. Indeed, enhanced connectivity has been observed during mindfulness and other cognitive tasks that involve distraction from pain (48, 49). Conversely, connectivity within the *limbic network* may facilitate the memorization of mechanosensory information in acute LBP, leading to behavioral modification and the memorization of the context (16, 50).

These results suggest an association between body awareness and dysfunctional sensory and nociceptive processing, linked to central sensitization and associated components, such as the wind-up phenomenon. The findings emphasize the complex interplay between sensory processing and the brain's cognitive and emotional networks in pain states, as well as the crucial role of integrating sensory, cognitive, and emotional components. Understanding these connections could inform the development of targeted therapies, with cognitive interventions potentially modulating specific neural pathways to alleviate persistent pain (51, 52). A person-centered approach that reduces overall sensorimotor system excitability may be helpful. Further research should explore the relationship between the wind-up phenomenon, affective pain perception, and motor behavior.

#### Movement control impairment (MCI)

In an episode of LBP, MCI changes lumbopelvic kinematics. The following reduction in variation in spinal motion (53) and the increase in muscle tension add additional strain on the spine (54, 55). This study is the first to associate MCI test scores with adaptations across multiple brain networks. In the *salience network,* the insular cortex emerges as a key hub for the subsequent processing of sensorimotor input, enabling the integration of sensorimotor information with interoceptive awareness and affective and salience-related cues on the somatosensory side (56). Adaptation in motor programming may be facilitated by enhanced connectivity-modulation between the supramarginal gyrus and the supplementary motor area. This connectivity is well known for influencing internally generated movements, particularly the anticipatory postural control. Evidence from motor imagery and resting-state MRI studies has revealed enhanced connectivity between these brain areas in participants with impaired trunk motor control, larger repositioning errors, and increased co-contraction (57). Therefore, enhanced connectivity in these areas may indicate changes to the motor repertoire. Under normal conditions, the brain conserves energy as efficient neural activation is critical for effective movement control, sensorimotor learning, and motor adaptation (54).

Findings in the *sensorimotor networks* further support these finding*s*. Here, the connectivity-modulation between the primary motor cortex and inferior parietal areas, and its strong relationship with MCI, support, on the one hand, the hypothesis of actual trunk movement initiation during mechanosensory stimulation. Additionally, it strengthens the case for a change in the motor repertoire through enhanced somatosensory processing during movement execution.

Negative associations were found in the *limbic network*, specifically in connectivity-modulation between the parahippocampal and insular cortices. Both regions are critical for sensory and emotional processing. In the context of LBP, the integration of sensory and emotional experiences may influence the perception of pain and the memory of it, thereby affecting coping and pain management strategies. In acute LBP, the body's drive for behavioral adaptation aims to protect the lumbar spine from further tissue damage or pain, leading to changes in connectivity between brain regions involved in motor executive functions and in the storage of novel movement patterns. Clinically, changes in movement control can result in adaptive movement patterns, such as co-contraction, defensive muscle tensing, changes in activity patterns, or avoidance of trunk movements. Although multiple factors influence the development of MCI during acute LBP, interventions targeting movement control can effectively reduce the risk of persistent pain and disability.

### Strengths and limitations

It is important to consider how specific our results may be to participants with acute LBP. The findings are based on associations between online surveys and clinical assessments, respectively, and functional connectivity measures assessed within one week. Most findings support evidence often observed in daily clinical assessments and treatments, and they link these observations to brain correlates. However, the assessment of multiple factors was a strength; future studies should also consider examining variables during fMRI acquisition. Methodologically, we based our initial sample size on previous studies, which we acknowledge may be relatively small. Unfortunately, missing clinical test values reduced the analytical sample size, further limiting the statistical power to detect substantial effects. The 18–60 age range introduces variance from ongoing early adult neurodevelopment and early middle-age network reorganization. Because age was not included as a covariate, this variance may influence connectivity analyses. Therefore, our results should be interpreted with caution. Finally, the computation of associations between clinical variables and brain connectivity modulation is not straightforward so that interpretations may be open to speculation.

## Conclusion

This study examined mechanosensory stimulation of the lower back using seed-to-whole-brain functional connectivity with predefined networks in individuals with acute LBP, and its relation to previously collected psychometric and clinical data. Despite various psychometric assessments, only pain intensity and avoidance behavior correlated with specific brain networks. Our results revealed significant associations among wind-up ratio, MCI tests, and modulation of brain connectivity, suggesting widespread sensorimotor adaptation during acute LBP episodes. The results support the idea that early connectivity changes involving sensorimotor and cognitive-behavioral processes reflect adaptation in response to acute LBP. The research highlights the importance of understanding the neural mechanisms underlying sensory processing and motor control in acute LBP. It provides potential avenues for targeted interventions to reduce the risk of persistent pain and disability from a biopsychosocial perspective. Furthermore, it may add a piece of the puzzle in understanding the factors involved in the transition from acute pain to a chronic or recurrent stage of LBP.

## Data Availability

The raw data supporting the conclusions of this article will be made available by the authors, without undue reservation.
